# Overexpression of rod photoreceptor glutamic acid rich protein 2 (GARP2) increases gain and slows recovery in mouse retina

**DOI:** 10.1186/s12964-014-0067-5

**Published:** 2014-10-17

**Authors:** Shanta Sarfare, Alex S McKeown, Jeffrey Messinger, Glen Rubin, Hongjun Wei, Timothy W Kraft, Steven J Pittler

**Affiliations:** Department of Vision Sciences, University of Alabama at Birmingham, Birmingham, AL 35294-0019 USA; Current address: Department of Ophthalmology, College of Medicine, University of Florida College of Medicine, Gainesville, Florida USA; Department of Ophthalmology, University of Alabama at Birmingham, Birmingham, AL 35294-0019 USA; Impulse Monitoring, Inc., 10420 Little Patuxent Pkwy, Suite 250, Columbia, Maryland 21044 USA; Current address: Department of Pathology, Division of Molecular and Cellular Pathology, Birmingham, AL 35294 USA

**Keywords:** Retina, Rod photoreceptor, *Cngb1a*, cGMP-gated cation channel, Phototransduction

## Abstract

**Background:**

The rod photoreceptor cGMP-gated cation channel, consisting of three α- and one β subunit, controls ion flow into the rod outer segment (ROS). In addition to the β-subunit, the *Cngb1* locus encodes an abundant soluble protein, GARP2 that binds stoichiometrically to rod photoreceptor cGMP phosphodiesterase type 6 (PDE6). To examine the *in vivo* functional role of GARP2 we generated opsin promoter-driven transgenic mice overexpressing GARP2 three-fold specifically in rod photoreceptors.

**Results:**

In the GARP2 overexpressing transgenic mice (tg), the endogenous channel β-subunit, cGMP phosphodiesterase α-subunit, peripherin2/RDS and guanylate cyclase I were present at WT levels and were properly localized within the ROS. While localized properly within ROS, two proteins cGMP phosphodiesterase α-subunit (1.4-fold) and cGMP-gated cation channel α-subunit (1.2-fold) were moderately, but significantly elevated. Normal stratification of all retinal layers was observed, and ROS were stable in numbers but were 19% shorter than WT. Analysis of the photoresponse using electroretinography (ERG) showed that tg mice exhibit no change in sensitivity indicating overall normal rod function, however two parameters of the photoresponse significantly differed from WT responses. Fitting of the rising phase of the ERG a-wave to an accepted model of phototransduction showed a two-fold increase in phototransduction gain in the tg mice. The increase in gain was confirmed in isolated retinal tissue and by suction electrode recordings of individual rod photoreceptor cells. A measure of response recovery, the dominant time constant (τ_D_) was elevated 69% in isolated retina compared to WT, indicating slower shutoff of the photoresponse.

**Conclusions:**

GARP2 may participate in regulating visual signal transduction through a previously unappreciated role in regulating phototransduction gain and recovery.

**Electronic supplementary material:**

The online version of this article (doi:10.1186/s12964-014-0067-5) contains supplementary material, which is available to authorized users.

## Background

Light perception is initiated by the process of phototransduction that occurs in the outer segments of rod and cone photoreceptors [1]. Rods have much higher light sensitivity, lower “dark noise” and higher phototransduction gain compared to cones [2,3]. In rods, the activation of the visual pigment rhodopsin by a photon of light initiates a cascade of events, involving activation of the G-protein, transducin, leading to hydrolysis of cytoplasmic cGMP by cGMP phosphodiesterase type 6 (PDE6). PDE6 activation occurs through removal of an inhibitory constraint imposed by the enzymes’ own γ-subunits. The cGMP-gated cation (CNG) channels close in response to reduced cGMP binding, causing a transient hyperpolarization of the membrane voltage resulting in a reduction of glutamate release at the synapse. Recovery of the photoresponse requires a series of reactions that returns the photoreceptor to the dark state. Rhodopsin activity is shut off through phosphorylation and arrestin binding, transducin through regulated intrinsic GTPase activity, and PDE6 through reestablished inhibition by its γ-subunits [1].

The CNG channels are critical in converting the chemical events during light activation into an electrical signal, which is relayed to the bipolar cells [4,5]. The rod CNG channel is a heterotetramer consisting of 3 α (CNGA1) and 1 β (CNGB1) subunits arranged around a central pore [6-8]. The CNGB1 locus encoding the channel β-subunit consists of 33 exons and has a bipartite structure comprised of an amino-terminal glutamic acid-rich (GARP) region and a carboxy-terminal channel-like region [9-11]. In rod photoreceptors, via alternative splicing, the GARP region of the CNGB1 locus is also expressed as two shorter soluble proteins. One of these proteins, GARP2 is a 32 kDa protein and is about 20-fold more abundant than the other soluble protein, GARP1. Both proteins are so named because they are very rich in glutamic acid (pI ~ 4) and they also contain a high concentration of proline residues [10]. All three proteins show anomalous decreased migration in SDS gels due to the highly negatively charged GARP region [12]. The high proline content contributes to the proteins being intrinsically disordered, which can allow for structural flexibility and binding to multiple partners according to the physiological milieu [13]. GARP2 is expressed only in rods [12,14,15], where it binds peripherin/RDS [16] and PDE6 with high affinity [17,18]. A study also indicates that GARP2 can bind to the cGMP-gated cation channel complex and inhibit channel gating thereby reducing dark noise [15]. GARP2 suppresses PDE6 activity in the dark through binding to the polybasic region of the PDE6 γ-subunit [18,19], leading to the hypothesis that GARP2, through PDE6 interaction may also increase the efficiency of phototransduction by reducing dark noise [18]. In contrast, excess PDE6 γ-subunit causes a reduction in phototransduction gain, but a more rapid recovery of the photoresponse [20]. In this study, we have found an opposite effect of excess GARP2 in mouse rods: increased gain and slowed recovery. These results indicate a novel role of GARP2 in phototransduction where it acts to regulate phototransduction gain and response recovery.

## Results

### GARP2 transgenic mice overexpress GARP2 in the rod photoreceptors

**Figure 1:**
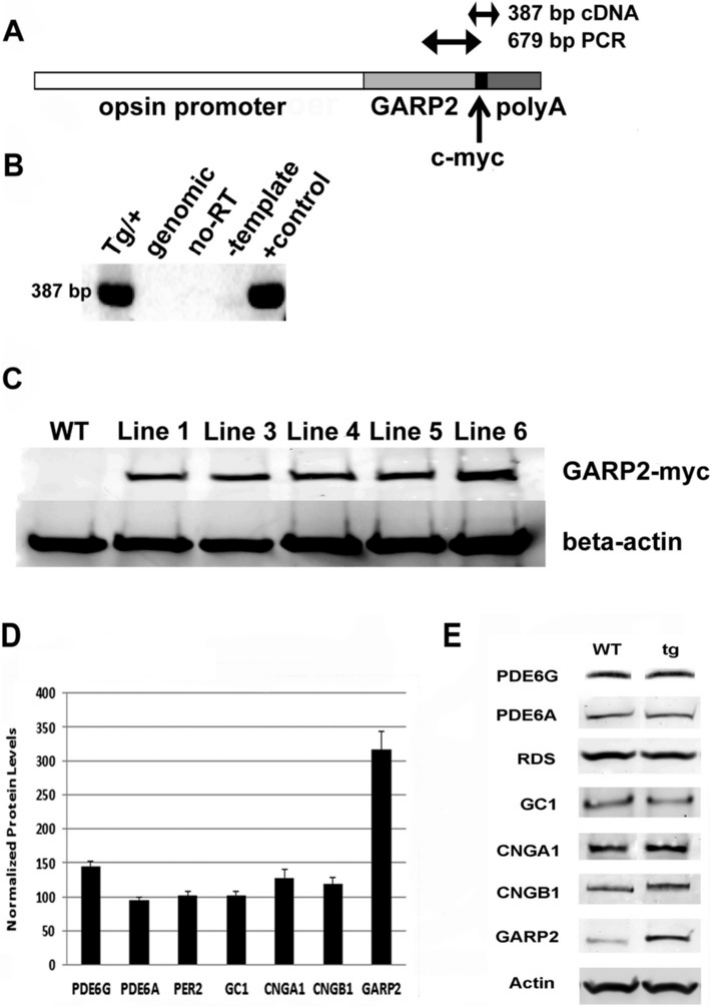
**Transgenic mice expressing GARP2 in the rod photoreceptors.** Schematic representation of the GARP2 transgene. **(A)** tg cDNA driven by a 4.8 kb mouse opsin gene promoter, with a c-myc tag added near the C-terminus of GARP2 and mouse protamine polyA signal. The relative locations of the region of the transgene amplified by PCR for genotyping (679 bp) and RT-PCR (387 bp) are denoted by double headed arrows. **(B)** Reverse transcription of transgene-specific transcripts in tg retina. Total RNA was reverse transcribed and PCR amplified with a primer pair specific for the tg and the myc tag insertion sequence. The expected 387 bp PCR product was only observed in mice carrying the tg (Tg/+) and a control template sample (+control), but was not amplified from genomic DNA (genomic), in the absence of reverse transcriptase (no-RT) or in the absence of template DNA (−template). **(C)** Western blot with a myc tag antibody showing tg protein expression in the retinas of 5 transgenic lines and absence in WT. Relative levels of ROS proteins in GARP2 transgenic mice. **(D)** Quantitation of ROS protein levels normalized to 100% WT. **(E)** Representative Western blots showing relative levels of ROS proteins PDE6G (n = 3), PDE6A (n = 3), PER2 (n = 4), GC1 (n = 3), CNGA1 (n = 4), CNGB1 (n = 3) and GARP2 (n = 6) in tg compared to WT. CNGA1 was increased 30% (P < 0.01). Total GARP2 expression was increased 3-fold (P < 0.001), PDE6G was elevated 1.4-fold, and other ROS proteins were unchanged.

To investigate the role of GARP2, we developed a transgenic mouse model overexpressing GARP2 in the rod photoreceptors. Transgene [21] expression was directed specifically to the rod photoreceptors using a 4.4 kb opsin gene promoter [22], and a mouse protamine gene polyadenylation signal was used for proper expression of the transgene mRNA [23] (Figure 1A). Five independent founder lines designated 1, 3, 4, 5 and 6 were generated from the GARP2 transgene construct.

To verify mRNA transcription from the transgene, we performed RT-PCR on retina cDNA from the transgenic mice using transgene-specific primers. A product of 387 bp was generated by PCR (Tg/+, Figure 1B) of the transgene-containing plasmid used for microinjection. The antisense primer is specific to the c-myc region, and thus the product is specific to the transgene transcript. PCR products were not generated from genomic DNA (Genomic), in the absence of reverse transcriptase (No-RT), or in the absence of template (−Template). The same size product was obtained with RT-PCR from Line 3 transgenic retina cDNA, demonstrating that the GARP2 transgene-specific mRNA was transcribed (Figure 1B).

To allow specific detection of GARP2 transgene expression a c-myc tag was included near the C-terminus. As shown in Figure 1C, using a c-myc tag-specific monoclonal antibody in Western analysis a single band of ~73 kD was detected from protein extracted from the retina from all five transgenic mouse lines. This band was not detected in the wild type (WT) mouse retina. We observed differing levels of expression of the GARP2-myc protein in the transgenic lines. Line 6 demonstrated the highest expression level (two-fold compared to other lines) and was used for all subsequent experiments.

### Some ROS protein levels are elevated in tg retina

To determine expression levels of GARP2 and other ROS proteins involved in cyclic nucleotide metabolism we performed quantitative Western analysis (Figure 1D,E). Immunoblotting with an N-terminal antibody that recognized GARPs and the channel β-subunit revealed single bands of the expected size for each protein (Figure 1E). The GARP2 transgene is expressed 3-fold over the WT levels in the transgenic retina, PDE6G showed a 1.45-fold increase in expression and the cGMP-gated cation channel β-subunit (CNGA1) that can bind GARP2 [15] showed a small but significant increase. The levels of expression of PDE6A and peripherin2/RDS (PER2), two other proteins known to interact with GARP2, the channel β-subunit (CNGB1) and guanylate cyclase 1 (GC1) levels remained unchanged (Figure 1D). Thus, GARP2 overexpression did not affect expression of most ROS proteins examined.

### ROS length is reduced in tg, but ROS width, ONL thickness remain unchanged

**Figure 2:**
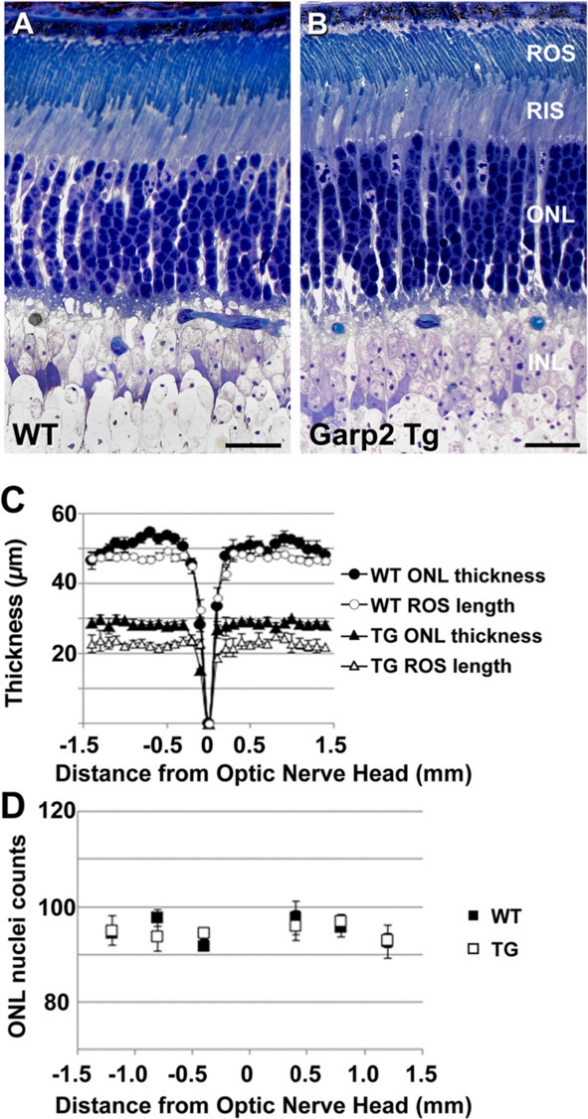
**GARP2 transgene overexpression does not affect gross retinal morphology.** Cross section through the retina of 1 mo WT **(A)** and tg **(B)**. All retinal layers appear normal in the tg at 1 month (Scale bars, 20 μm). Morphometric analysis of the tg retina at 1 month **(C, D)**. Spider graph plot in **(C)** shows differences in thickness of ROS in tg (n = 4) and WT (n = 5) and outer nuclear layer (ONL) at different eccentricities from the optic nerve head. **(D)** Average counts of ONL nuclei in WT and tg at specified distances from the ONH. No significant differences in total nuclei counts were observed.

At one month, retinal stratification in the transgenic mice appeared normal compared to age-matched wild type controls (Figure 2A,B) and no retinal degeneration was observed up to one year examined. To assess the integrity of the tg retina, we measured ROS length, outer nuclear layer (ONL) thickness, and total ONL nuclei counts (Figure 2C–D). ROS length in tg mice was reduced across most of the expanse of the retina (tg, Δ vs WT, ▲; Figure 2C), while ONL thickness was unchanged. ONL cell counts have been used as a quantitative measure of photoreceptor degeneration [24]. Fine measurements of ROS length and width were performed with ultra-thin sections imaged on an electron microscope. The average ROS length in WT mice was 27 ± 1.8 μm, which is in agreement with ROS length measurements from similarly processed tissue samples from adult C57BL/6 mice [25]. The average length of tg ROS at 1 month was 21.9 ± 1.8 μm, reflecting a 19% reduction in ROS length (p < 0.001). No difference in ROS width between WT (1.311 ± 0.117 μm) and tg (1.317 ± 0.116 μm) mice was observed (p > 0.5). The width measurements were in good agreement with measurements obtained from WT rods (1.32 μm) by cryoelectron tomography [26]. We observed no significant difference (P values ranged from 0.12 to 0.93 in each group) in the total number of ONL nuclei in a 250 μm^2^ area, measured in the plane of the optic nerve (Figure 2D). These results suggest that overexpression of GARP2 does not affect gross morphology of the retina and rod outer segments.

### GARP2 and other ROS proteins are properly localized in the transgenic retina

**Figure 3:**
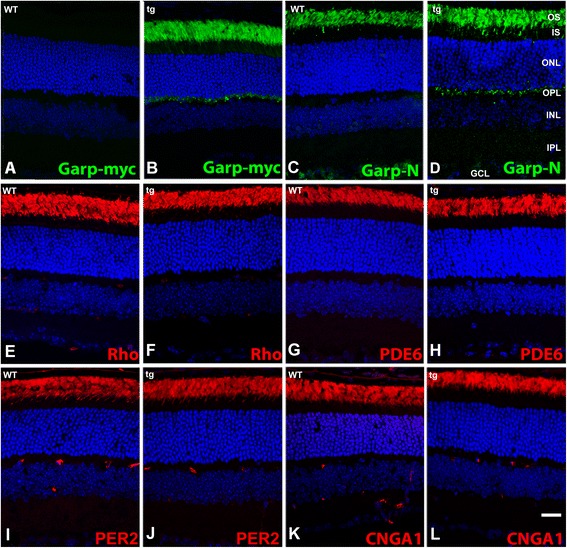
**Immunolocalization of GARP2 and other ROS proteins.** Myc tag epitope staining in wildtype **(A)** and GARP2 tg **(B)**. GARP localization in WT **(C)** and transgenic retina **(D)** with an N-terminal Garp/β-subunit antibody. Panels **E**, **G**, **I**, **K** are from WT retina, and **F**, **H**, **J**, **L** are from tg retina. Immunolocalization of rhodopsin **(E and F)**, PDE6 **(G and H)**, PER2 **(I and J)** and CNGA1 **(K and L)**. Nuclei are stained with DAPI. Scale bar in P, 20 μm.

To assess the spatial distribution of ROS proteins in the transgenic mouse compared to WT we performed immunohistochemistry (Figure 3). Using a myc-tag specific antibody tg expression was seen predominantly in the photoreceptor outer segment layer in transgenic mice, and as expected, transgene expression was not observed in non-transgenic WT retina (Figure 3A,B). A weak distribution of signal was also seen in the outer plexiform layer, as has been observed previously in WT retina with other GARP antibodies [12,14]. Labeling with an N-terminal common GARP antibody (3C, D) showed that GARP proteins and the β-subunit are localized to the ROS layer in the transgenic retina, thus demonstrating that GARP2 overexpression does not affect the distribution of other GARP-region containing proteins. The proper distribution of endogenous GARP2 was confirmed using a C-terminal GARP2 specific antibody that recognizes both endogenous and transgene-expressed GARP2 (Additional file 1: Figure S1). We also investigated the localization of endogenous ROS proteins that interact with GARP2 or are involved in phototransduction. Overexpression of GARP2 did not affect the normal outer segment localization of rhodopsin (Rho, Figure 3E,F), cGMP phosphodiesterase (PDE6, Figure 3G,H), peripherin2/RDS (PER2, Figure 3I,J) or the cGMP-gated cation channel α-subunit (CNGA1, Figure 3K,L).

### tg mice exhibit increased phototransduction gain *in vivo*

We initially assessed retinal function in 1 mo tg mice using dark-adapted electroretinography (ERG). The average maximum a-wave amplitude of WT mice was 657 ± 142 μV (n = 11), whereas that of the tg was 443 ± 57 μV (n = 11), a 33% reduction in maximum a-wave response (p < 0.001), which is consistent with the reduction in ROS length (Additional file 2: Figure S2). The mean b-wave amplitude was reduced from 1508 ± 411 μV for the WT to 1079 ± 103 μV in the tg, which is not a statistically significant difference (p > 0.05). Implicit times for the a-wave and b-wave were not significantly different between WT and tg animals for dark adapted saturating stimuli (a-wave: 16 ± 0.5 ms WT vs. 16 ± 0.6 ms tg; b-wave: 73 ± 3 ms WT vs. 70 ± 2 ms tg; p > 0.05) or for light adapted stimuli (a-wave: 13 ± 1 ms WT vs. 14 ± 1 ms tg; b-wave: 54 ± 1 ms WT vs. 53 ± 1 ms tg; p > 0.05, WT n = 8; and tg n = 7). Intensity response curves fit with equation 1 for both a-wave and b-wave response amplitudes are nearly overlapping and the I_1/2_-values are not significantly different, indicating no change in sensitivity of the tg mice (p > 0.05, Additional file 2: Figure S2).

**Figure 4:**
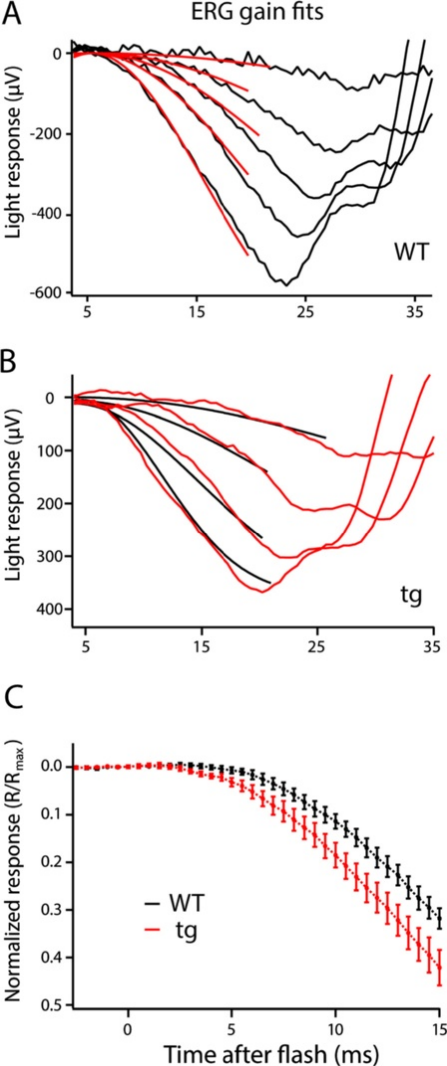
**Analysis of phototransduction gain in live mice. (A)** An example fit of the rising phase of the a-wave in ERG responses in WT mice for a range of 1,400-44,000 photon/μm^2^ incident at the cornea. Individual electrophysiological responses are shown in black with the global gain fits overlaid in red. **(B)** Example fit of the rising phase of the a-wave in ERG responses to a flash range of 2,300-52,000 photons/μm^2^ incident at the cornea in a tg mouse. Individual electrophysiological responses are shown in red with the global gain fits overlaid in black. **(C)** Response amplitudes for flashes of 48,000 photons/μm2 incident at the cornea were normalized to the R_max_ for each individual animal to illustrate the difference in the rising phase of the a-wave. WT (black, n = 8) and tg (red, n = 7).

To characterize the activation phase of phototransduction, we fit the a-wave data to a model of rod phototransduction (Equation 2, described in Methods) [27,28]. Five different a-wave responses to 10 ms flashes of different light intensities were simultaneously fit to Equation 2, and the curves best fitting the equation were used to obtain estimates of phototransduction gain in WT and tg mice (Figure 4A-B). Figure 4C shows the rising phase of the average a-wave response to flash intensity corresponding to 48,000 photons/μm^2^ incident at the cornea (WT n = 8; tg n = 7). Using the ensemble fit for all a-waves, we obtained values of A_g_ = 6.7 ± 0.6 s^−2^ for the WT and A_g_ *=* 13.9 ± 1.4 s^−2^ for the tg (Table 1). The A_g_ parameter is increased 2-fold (105%) in the tg mice (p < 0.001), which indicates an acceleration in the activation phase of the rod photoreceptor light response. Significantly increased estimates of A_g_ were also obtained when the gain analysis was repeated using 2 ms flash duration, to better approximate an impulse. The curve fitting analyses was performed in two different ways: (1) using observed values of a_max_, and (2) using a_max_ as a fixed calculated ratio of the response amplitude to non-saturating light levels to avoid b-wave contamination. For both of these fitting algorithms, significantly increased values of A_g_ in the tg were obtained. Two additional independent methods of analysis, isolated tissue ERG and single cell suction electrode recordings were used to avoid complications from b-wave intrusion.

**Table 1:** **Response properties and gain analysis for rods from WT and GARP2 tg mice**

	**Genotype (n)**	**R**_**max**_	**I**_**1/2**_ (R*)	**spr**	**T**_**i**_ **(ms)**	**T**_**p**_ **(ms)**	**τ**_**D**_ **(ms)**	**Gain (s** ^**−2**^ **)**
**Live ERG**	WT (8)	585 ± 158 μV						6.7 ± 0.6*
	tg (7)	398 ± 93 μV						13.9 ± 1.4*
**Tissue ERG**	WT (21)	241 ± 25 μV	55 ± 5				99 ± 5 *	3.5 ± 0.5*
	tg (14)	181 ± 26 μV	59 ± 9				167 ± 17*	6.6 ± 0.7*
**Single cell**	WT (29)	12.6 ± 0.7 pA	21.7 ± 1.5*	0.45 ± 0.02	312 ± 17	152 ± 6*	160 ± 6.9*	7.9 ± 0.6 (n = 20)*
	tg (13)	11.2 ± 0.6 pA	13.0 ± 2.0*	0.42 ± 0.05	277 ± 24	131 ± 5*	209 ± 11.5*	16.5 ± 2.2*

*Indicates significance (p < 0.05).

Criterion for inclusion in the data set was a maximum response (R_max_) of at least 7 pA. Phototransduction gain analysis only includes cells whose single photon response had been determined, thus defining the collecting area of the cell (see Methods). Values are shown as mean ± SEM. Labels are, half-maximal intensity, (I_1/2_), single photon response amplitude (spr), Integration time (T_i_), time-to-peak of linear range responses (T_p_), dominant time constant of recovery, (τ_D_), and phototransduction gain (Gain).

t-test p-values:

(WT vs tg) Single cell gain: 0.0000484 Single cell τ_D_: 0.000365 T_p_: 0.0267.

Tissue gain: 0.000695 Tissue τ_D_: 0.0000658.

### Rods overexpressing GARP2 exhibit increased phototransduction gain *in vitro*

**Figure 5:**
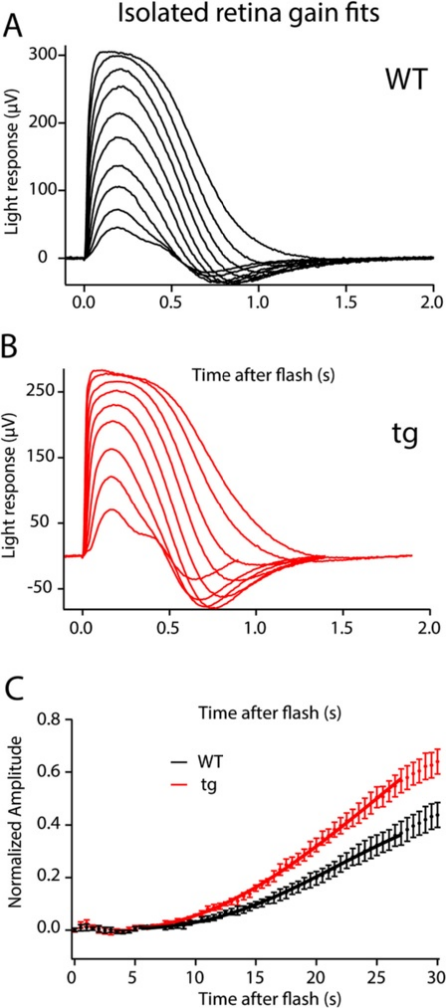
**Analysis of gain in isolated retinal tissue. (A)** An example WT retina in the isolated tissue ERG preparation. Stimulus range was 4–1350 R*/flash. **(B)** An example tg retina recorded in the isolated tissue ERG preparation. Stimulus range was 18–980 R*/flash. **(C)** Response amplitudes for flashes eliciting 260 R*/flash in both WT (black) and tg (red). Responses were normalized to the R_max_ for each individual retina to illustrate the difference in the rising phase of the rod photoresponse; WT (black, n = 21) and tg (red, n = 14).

To confirm the observed increase in gain we analyzed isolated retinas that were pharmacologically manipulated to eliminate inner retina responses, allowing analysis of the photoreceptor fast PIII response (Figure 5A-B; Table 1) [29,30]. Figure 5C shows a comparison of gain estimates to the rising phase of the a-wave in WT and tg isolated retinas using Equation 2 (see Methods). The tg mice retinas show an 89% increase in gain (WT, 3.5 ± 0.5 s^−2^; tg, 6.6 ± 0.7 s^−2^; p < 0.001) in agreement with the *in vivo* estimates of phototransduction gain for the *in vivo* ERG a-wave analysis.

**Figure 6:**
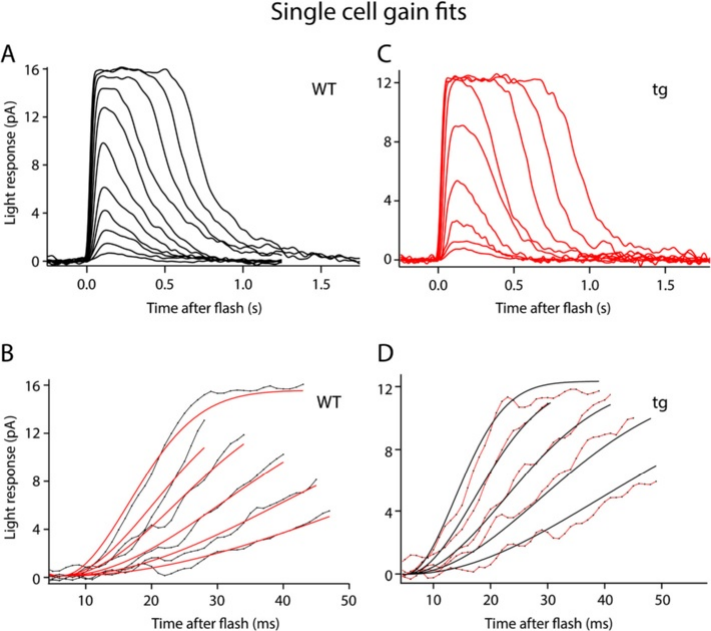
**Analysis of gain in single rod photoreceptors. (A)** A family of responses from an exemplary WT rod, with stimuli activating 0.4-660 R*/flash. **(B)** Rising phases of the cell in **(A)** representing the global fits estimating gain using equation (3). Gain for this cell was 5.8 s^−2^. **(C)** A family of responses from a tg rod with stimulating activity 1.3-615 R*/flash. **(D)** Rising phases of the cell in **(C)** representing the global fits estimating gain using equation (3). Gain for this cell was 17.3 s^−2^.

**Figure 7:**
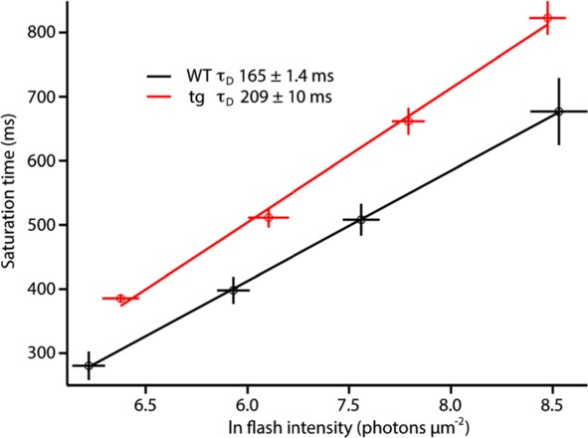
**Pepperberg plots of the dominant time constant of recovery (τ**_**D**_ **) for single cell recordings.** By plotting saturation time at 75% recovery against the ln of photon density, the slope of a linear fit reveals the dominant time constant of recovery. Each point represents the average of WT n = 29 and tg n = 13 cells. WT, τ_D_ = 165 ± 1.4 ms and tg, τ_D_ = 209 ± 10 ms.

In order to further evaluate the properties of the tg rods and to confirm the increase in gain by a third method, single cell suction electrode recordings were performed (Figure 6; Table 1). Under IR illumination the outer segment of a rod was drawn into an electrode to record the circulating dark current and the responses to light. Several properties of the individual rods were recorded and found to be different in WT compared to tg rods. The half-maximal R* levels (I_1/2_) were calculated from the full range of response amplitudes, and were significantly different between WT and tg (p < 0.002) consistent with the increase in phototransduction gain. Shown in Figure 6B and 6D are representative curve fittings used to analyze gain in a single WT and tg rod, respectively. For 13 cells whose collecting area was experimentally calculated, phototransduction gain was elevated two-fold in the tg mice (WT, 7.9 ± 0.6 s^−2^, tg 16.5 ± 2.2 s^−2^, p < 0.0001). As shown in Table 1, three parameters in addition to the gain; T_p_, the time-to-peak of the linear range response, I_1/2_, the sensitivity measure of the cell and, (τ_D_), the dominant time constant, a measure of response recovery time, were significantly altered in rods from transgenic animals. τ_D_ was significantly elevated in both isolated tissue (τ_D_, 69% increase; p < 0.0001) and in single cells (τ_D_, 31% increase, p < 0.001). The increase in τ_D_ indicates a slower shut off of the phototransduction cascade. In WT animals τ_D_ represents the inactivation rate of PDE6, but it can be altered due to a variety of molecular perturbations [20,31-33]. Pepperberg plots [34] indicate the average τ_D_ values (linear slope) calculated from WT and tg single cells (Figure 7). The time-to-peak (T_p_), and integration time (T_i_) were measured from linear responses. The magnitude of the spr estimates were not significantly different for the individual data (0.42 ± 0.07 pA tg, 0.45 ± 0.02 pA WT; p > 0.28). However, the T_p_ values revealed that the tg rods peaked faster than the WT (131 ± 5 ms tg, 152 ± 6 ms WT; p < 0.05) but T_i_ did not differ. The T_p_ result is consistent with the findings from analysis of saturating responses, which indicated an increase in gain of the light response (Table 1).

## Discussion

In this study we have investigated the functional consequence of overexpression of GARP2 in the rod photoreceptors. Although *in vitro* models suggest that GARP2 may play a role in increasing rod sensitivity in the dark [18], the *in vivo* function of GARP2 had not been established. While morphometric analysis showed that the average length of the tg ROS was reduced, there were no other gross changes in retinal health, as the number of ONL nuclei in the tg were not statistically different from WT. However, some features of phototransduction were altered in tg mice when compared to WT: phototransduction gain was increased, the dominant time constant of recovery was longer, and linear range responses peaked earlier. The sensitivity of the tg rods was also significantly higher (as demonstrated by a lower I_1/2,_ Table 1) than WT when measured by single cell recordings. In the whole animal ERG or isolated tissue ERG I_1/2_ was not found to differ, however sensitivity measures in single cells are generally more accurate and reproducible from cell to cell. Thus, we conclude that the tg rods are more sensitive than WT rods, consistent with the finding of greater phototransduction gain. It is possible that a single property of GARP2 can explain each of these outcomes. We propose a model in which GARP2 influences PDE6 activity by *a)* reducing basal PDE6 activity in the dark and *b)* decreasing the rate of inactivation of active PDE6 during recovery from a light response.

Modulation of phototransduction gain may occur due to a change in any of the biochemical steps of activation (for a review see [35]). We observed reduction in ROS length, but no change in ROS width, or in the levels of several ROS proteins in the tg retina. We also examined rod disk spacing (Additional file 3: Figure S3) and performed quantitative spectrophotometric determination of rhodopsin levels and found no significant differences (WT, 26.35 μg/mg total protein; tg, 26.32 μg/mg total protein; T-Test p > 0.99; ANOVA WT vs tg F(1,11) = 5.32, p > 0.73 rhodopsin vs total protein F(1,11) = 5.32, p > 0.18). Thus, the ROS volume is 19% smaller but protein levels are constant meaning the disks will be crowded with 19% more protein, mostly rhodopsin, extra GARP2 and PDE6G.

The basal rate of PDE6 activation is determined by the state of its two γ-subunits. In darkness, the γ-subunits are in an inhibitory confirmation. When rhodopsin activates transducin-α (T*), T* then displaces the PDE6 γ-subunits to activate PDE6 catalytic activity. In darkness, the γ-subunits can spontaneously change conformation without transducin, which also allows the PDE6 enzyme to hydrolyze cGMP. In an *in vitro* experiment, WT GARP2 was shown to silence 80% of the basal activity of PDE6 in the dark [18], by binding to the PDE6 γ-subunit [19]. Also, GARP2:PDE6 stoichiometry was determined to be 1:1 in WT rods [18]. A 1:1 ratio suggests that GARP2 silences one γ-subunit at a time, allowing the other subunit to spontaneously activate the enzyme. The exquisite sensitivity of rods is achieved due to a very low level of background, or “dark noise” [36,37]. The continuous aspect of this noise is generated in part by a basal level of spontaneous activity of PDE6 [38]. Pentia et al. [18] have proposed that GARP2 can reduce this noise in rods by quieting PDE6 through an interaction with the PDE6 γ-subunit. In the transgenic rods, excess GARP2 could be inhibiting a larger proportion of the PDE6 γ-subunits, thus decreasing spontaneous PDE6 activation. Such an interaction would decrease the frequency of fluctuations in cGMP levels and reduce the continuous noise component in the cells. It is unknown if such a decrease in noise would translate to the increase in gain that we detected in this study. However, lower basal PDE6 activity could facilitate a faster activation phase from T* simply by increasing the probability that T* would collide with a completely inactive PDE6 molecule. The concomitant increase in levels of PDE6 γ-subunit determined in this study with elevated GARP2 (Figure 1D,E) are consistent with a proposed role for PDE6 γ-subunit/GARP2 interaction [19]. Thus, the actual effect of GARP2 on increasing gain could have been more pronounced, if PDE6 γ-subunit levels were not also elevated. Excess PDE6 γ-subunit in transgenic rods by itself reduces phototransduction gain [20], but the GARP2 to PDE6 γ-subunit ratio in our transgenic rods is strongly tilted in favor of GARP2 and is thus consistent with the idea that excess GARP2 increases phototransduction gain.

Altered disk spacing or changes in the levels of phototransduction proteins, could affect phototransduction gain [39]. To address disk spacing we measured the spacing between disks in high magnification TEM images in WT and tg mice and found no difference in spacing between disks (Additional file 3: Figure S3), consistent with disk spacing not being a factor in the observed increase in gain. Another possible mechanism is GARP2 channel gating inhibition reducing spontaneous channel openings [15]. This interaction could be important in reducing dark noise in the tg mice as more GARP2 would be available for channel binding leading to increased gain. As yet, the exact mechanism for the increase in gain and the relative roles of PDE6 and channel inhibition by GARP2 remains unknown.

Our second significant finding was that overexpression of GARP2 increased the dominant time constant of recovery (τ_D_). Decreased rhodopsin concentration was reported to lead to faster response recovery [40], suggesting that the opposite, increased rhodopsin, could slow recovery. While we found no change in rhodopsin levels measured spectrophotometrically and normalized to total retina protein, it may be that increased rhodopsin density in the disk could play a part in extended R* lifetime.

The rate limiting step of saturated photoresponse recovery has been determined to be inactivation of T* by the Regulator of G-protein Signaling (RGS) complex [32,34]. The RGS complex catalyzes the conversion of T*-GTP to T*-GDP, which allows the γ-subunits of PDE6 to reinstate their inhibition, the step that ultimately turns off the catalytic activity of PDE6 allowing the cyclase to restore cGMP levels and thus end the light response [41]. Based on the established interaction between GARP2 and PDE6 [18,19], the overabundance of GARP2 may be interfering with the rate at which the RGS complex can shut off T* in the activated PDE6 complex. Alternatively, excessive GARP2 binding to the γ-subunits may slow the rate at which they re-associate with the PDE6 catalytic subunits. In either case, GARP2 is likely to be influencing PDE6 shutoff in WT rods. Consistent with this hypothesis, PDE6 γ overexpression shows a reduction in τ_D_, the opposite effect from what we observed with GARP2 overexpression (WT: τ_D_ = 205 ms; rods overexpressing PDE6 γ-subunit: τ_D_ = 91 ms [20]).

The rod photoresponse is also regulated by calcium feedback pathways [42]. Remarkably, due to their charged nature, GARP proteins can bind millimolar levels of Ca^2+^ with low affinity [43], and therefore may be involved in regulating intracellular calcium levels. It is tempting to propose a model where excess GARP2 in the transgenic rods may be acting as a Ca^2+^ buffer, thus affecting the Ca^2+^ feedback pathways. However, without actual measurement of internal calcium changes in response to light, precise conclusions cannot be made.

## Conclusions

In summary, the striking finding of this study is that GARP2 expression can influence phototransduction activation and recovery. For whole animal ERG, fitting the rising phase of the a-wave to the rod phototransduction model [27,44] showed an increase in the gain or efficiency of phototransduction. This result was confirmed in isolated retina ERG and in suction electrode recordings of single cells. Our findings establish a role for GARP2 in the phototransduction cascade where it regulates response kinetics, likely through its interaction with PDE6. Further studies are underway to further clarify the role of GARP2 through the generation of a rod specific GARP2 knockout mouse.

## Methods

### Production of GARP2 transgenic mice

All animal procedures were approved by the Institutional Animal Care and use Committee of the University of Alabama at Birmingham (UAB). Transgenic mice were generated in the Embryonic Stem Cell and Transgenic Core Facility at UAB. First strand cDNA was isolated from WT mouse retina as described in the next section. GARP2 cDNA was PCR amplified from the first strand cDNA using the following primer pairs: *Forward 5*′-AGTCCTCGAGAGCTGCTGGGGCACTGAGAAGG-3′ and *Reverse* 5′-TGCATGATCAGAGGCCTCCAGCTGCTGTCGCCCTCAGATCCTCTTCTGAGATGA-GTTTTTGTTCGGTCTGTACGTCACACATCCCAGGG-3′. A c-myc epitope sequence was engineered between GARP2 exons 12 and 12a to differentiate the transgenic from the WT GARP2 expression. The vector backbone used for generating the tg construct was assembled from the PDE6G tg construct [21] containing the 4.4 kb mouse opsin promoter [22] and the polyadenylation signal of the mouse protamine gene [23]. The PDE6G cDNA was excised using *BamHI* restriction enzyme and replaced with GARP2 cDNA using compatible restriction sites. The GARP2-myc coding region was bi-directionally sequenced using Applied Biosytems automated DNA sequencing at the core facility of the UAB Heflin Center for Genomic Science. The tg was excised from the plasmid backbone using *KpnI* and *XbaI,* purified with QIAquick Gel extraction kit (Qiagen), microinjected into C57BL/6 J mouse embryos and implanted into pseudo-pregnant females. Transgenic founder progeny were identified by PCR-based genotyping of tail genomic DNA. A 679 bp PCR product spanning the junction between the rhodopsin promoter and the transgene was used to identify the transgenic mice. Primers used were *Forward:* 5′-GCTGGTCCCAGCCTTCAAGAGA-3′ (SP3) and *Reverse:* 5′-CTCTTCTGAGATGAGTTTTTGTTCGGTC-3′ (ASP3). PCR was performed using an initial denaturation of 94°C for 5 min, followed by 35 cycles of amplification 94/62/72°C for 45/45/60s followed by a final extension at 72°C for 5 min. Founder mice were individually crossed to C57BL/6 J mice to establish separate transgenic lines. All mice used in the experiments were obtained by crossing a transgenic male with C57BL/6 J females. All experimental Garp2 transgenic mice were heterozygous for the transgene insertion; non-transgenic littermates were used as controls.

### RNA isolation and reverse transcription PCR

Total RNA was isolated from 1 month old mice (Versagene RNA tissue kit, Gentra Systems), digested with DNaseI and quantitated with ND-1000 spectrophotometer (Nanodrop, Thermo Scientific). Primer sequences were designed in-house with Vector NTI v.10 (Invitrogen). Briefly, 1 μg total RNA was reverse transcribed with Improm-II Reverse Transcriptase (Promega) using random decamers and oligo-dT primers. PCR was performed under standard conditions using 1–2 μl of five-fold diluted first strand cDNA. PCR primers spanned the GARP2-myc junction to distinguish transgenic transcripts from WT. The PCR reactions were repeated three times and analyzed by agarose gel electrophoresis.

*Generation of custom GARP polyclonal antibodies-* Garp-N antibody was generated in chicken against the most N-terminal 15 amino acids of human GARP protein. The antibodies were purified from the egg yolks using an IgY purification kit and used for immunoblot and immunolocalization. A GARP2 C-terminal specific antibody was generated in rabbit against the last 12 amino acids (Genscript USA, Inc.). Production bleeds were analyzed with ELISA. Serum from rabbit 463 was used in immunoblot and immunolocalization experiments.

### SDS-PAGE and immunoblot analysis

Retinas were homogenized in 10 mM Tris (pH 7.5) containing 0.5% Triton X-100 and Mammalian tissue Protease Inhibitor Cocktail (Sigma-Aldrich). Cellular debris was removed by centrifugation at 1000 × g for 10 minutes at 4°C and the supernatant was used for Western blotting. Protein concentration was determined using 2-D Quant kit (Amersham/GE Life Sciences) using bovine serum albumin as a standard. Equal amounts of protein from each sample was fractionated on 10% SDS-PAGE (Bio-Rad) and transferred to polyvinylidene difluoride membrane (Amersham/GE Life Sciences) using semi-dry or wet transfer apparatus (Bio-Rad). After blocking in 2% ECL Advance blocking agent (Amersham/GE Life Sciences), the membranes were incubated overnight at 4°C with primary antibodies rabbit polyclonal PDE6A (1:1000 dilution, AbCam), mouse monoclonal PDE6G (A-2; 1:200 dilution, Santacruz Biotechnologies), goat polyclonal PER2 (1:1000, Santacruz Biotechnology), goat polyclonal Guanylate cyclase 1 (1:1000, Santacruz Biotechnology), chicken polyclonal Garp-N (1:10000), mouse monoclonal CNGA1 (1:1000, from Robert Molday) or β-actin (1;10000, Genscript). Finally, the blots were incubated for 1 hour with the cognate horseradish peroxidase labeled secondary antibodies (Santacruz Biotechnology) and detected with ECL Advance Chemiluminescence detection kit (Amersham/GE Life Sciences). The blots were imaged with Typhoon Trio laser scanning imager (GE Life Sciences). Each experiment was performed at least in triplicate. Densitometric analysis of each stained band was performed with Image quant software (GE Life Sciences). After performing background correction, the data for each protein of interest were normalized to β-actin as a loading control. The results were averaged across at least three experiments. Statistical analysis and graphs were performed in Microsoft Excel. All data are shown as ± standard error.

### Spectrophotometric determination of rhodopsin concentration

Retina samples (WT, n = 3; tg n = 3) were maintained in the dark or under dim red light for the entire procedure. Mice were dark adapted overnight, eyes were enucleated, retinas dissected with care to minimize RPE contamination and placed in 200 μl 10% Chaps, mixed by repeated pipetting, 200 μl Chaps was added and the sample mixed again by repeated pipetting 10×. The solution was centrifuged 1.5 minutes at 15,000 rpm and kept in foil on ice to prepare a Varian Cary 50 Bio UV/Visible Spectrophotometer to measure the concentration. The absorbance at 500 nm of a 150 μl aliquot of each sample was determined and the concentration was calculated according to Beer-Lambert Law, *Α* = *εcl* where A is absorbance (as measured), ε is the extinction coefficient (42,700 cm^−1^, c is concentration (calculated), and *l* is the path length (1 cm). To normalize to total protein content, protein concentration was determined from the same samples used for rhodopsin analysis. From the 10% Chaps sample 1 μl was removed for protein assay using a commercial Bradford assay (Bio-Rad Protein Assay). Error was calculated as standard deviation and rhodopsin concentration and total protein groups were compared by two factor ANOVA in Excel 2010.

### Immunofluorescence and confocal microscopy

Mouse eyes were harvested in ambient light and fixed in 4% paraformaldehyde containing 5% sucrose in 0.1 M phosphate buffer (pH 7.4) for 4 hours and cryoprotected in 20% sucrose as described [45]. Sections of 8–10 μm thickness taken with CM3050S cryostat (Leica) were dried at room temperature, blocked and permeabilized in 0.1 M phosphate buffer containing 10% goat serum and 0.2% Triton X-100 for 30 minutes. Primary and secondary antibodies were diluted in labeling buffer (2.5% goat serum and 0.1% Triton X-100 in 0.1 M phosphate buffer). The sections were labeled overnight at 4°C with primary antibodies mouse monoclonal anti-c-myc (1:1000, Molecular Probes/Invitrogen); chicken polyclonal anti-Garp-N (1:1000); rabbit polyclonal anti-Garp-C (1:100); mouse monoclonal anti-rhodopsin (1:1000, Santacruz Biotechnology); rabbit polyclonal anti-PDE6G (1:100, Thermo Fisher); mouse monoclonal anti-PER2 (1:50, Robert Molday) and mouse monoclonal anti-CNGA1 (1:100, Robert Molday). The sections were then labeled with appropriately diluted goat anti-mouse, anti-rabbit and anti-chicken Alexa 488 (green) or Alexa 594 (red) secondary antibodies (Molecular Probes/Invitrogen). The sections were also incubated with chicken IgY, mouse and rabbit IgG and secondary antibodies alone at the same dilutions as that used with the primary antibodies to serve as controls. The slides were cover slipped with Ultracruz Mounting Medium (Santacruz Biotechnology) containing DAPI for nuclear localization and visualized with 40× 1.3 na and 63× 1.4 na oil immersion objectives on a Zeiss LSM 710 Inverted Confocal Laser Scanning microscope, software Zen 2008 4.7.2 (UAB High Resolution Imaging Facility).

### Light and transmission electron microscopy

Mouse eyes were enucleated following euthanasia with 5% isoflurane and decapitation. The eyes were oriented with Tissue marker dyes (Richard-Allan Scientific) and fixed in 2% paraformaldehyde; 2.5% glutaraldehyde in 0.1 M sodium cacodylate buffer for 1 hour. The anterior segment and lens was removed and secondary fixation was performed in 1% osmium tetroxide and 0.125% potassium ferrocyanide in 0.1 M sodium cacodylate buffer for 2 hours in dark followed by dehydration in a graded series of alcohol. The eyes cups were then transitioned to propylene oxide and embedded in Embed 812 resin (Electron Microscopy Sciences). Light microscope sections were cut at 0.8 μm thickness, stained with 0.1% Toluidine blue (Electron Microscopy Sciences) and photographed with Axioplan 2 upright microscope using Axiocam MRc5 digital camera and Axiovision 4.6.3 software (Zeiss). Ultrathin sections (80–90 nm) were mounted on copper grids (Electron Microscopy Sciences) and photographed with a JEOL 1200 electron microscope (JEOL).

### Morphometric and statistical analysis

Measurements were done according to Wu et al. [46] with minor modifications as follows. Thickness of ONL and length of ROS layers was measured from Toluidine blue stained 0.5 μm epon sections imaged on an Axioplan 2 microscope as noted above. Measurements were performed along equally spaced intervals from the optic nerve in Adobe Photoshop CS4. Morphometric analyses were performed on 1 month old WT (n = 5) and tg (n = 4) samples. ROS length was measured in ultrathin TEM samples from the distal end of the inner segment to the tip of the outer segment at 7500× magnification using ultrathin sections (WT, n = 112; tg, n = 121) rods. For these measurements, we selected regions that were half-way between the central and peripheral retina. Only ROS that were apparent from the base to the tip were measured, disregarding any that appeared twisted or obscured by other cells or debris. Outer segment widths were measured at 5000× in 10 separate fields for 3 tg (n = 327) and 2 WT (n = 237) 30 day old mice. Total numbers of nuclei were manually counted from 3 equally spaced 50 um^2^ regions in the photoreceptor nuclear layer, located at 0.5 mm intervals on either side of the optic nerve (WT n = 5; tg n = 4). Disk spacing across three disks was measured directly (WT, n = 4; tg, n = 5) on high magnification TEM images (75 K to 200 K) using imageJ. To measure disk spacing three non-overlapping regions from each image not containing the same disks (~75 to 100 μm^2^) were used to measure the distance across three disks from the beginning of the outer membrane of one disk to the end of the outer membrane of the third disk. Prior to measurement images were uniformly sharpened in Photoshop V. 6 and processed for measurement in ImageJ 64 by setting the set scale function based on the TEM scalebar. Measurements were exported to Excel 2010 for averaging and statistics. Measurements were only made in regions where each disk was clearly discernable and disk spacing was uniform in the measured region. Three different disk regions were measured in each image and averaged for WT (n = 4) and tg (n = 5). Average three disk distance was for WT 0.092 ± 0.008 μm, and for tg 0.086 ± 0.005 μm; P > 0.17. Statistical significance for all measurements was determined by unpaired t-test in MS Excel with error expressed as standard error of the mean (SEM) or standard deviation (SD) as noted in the text.

### Electroretinography

All mice were maintained on a 12/12 hr light/dark cycle in the UAB animal facilities. Wild type and transgenic mice (n = 8 each) were dark adapted overnight and anesthetized with ketamine (IVX Animal Health) at 90.9 μg/g and xylazine (Lloyd Laboratories) at 9 μg/g bodyweight. The corneas were anesthetized with 0.5% proparacaine (Bausch & Lomb); the pupils were dilated with 2.5% phenylephrine (Ocusoft Inc.) and 1% tropicamide (Alcon Laboratories). Mice were kept warm by placing on a 37°C heated gel pad (Braintree Scientific) inside a Faraday cage. Electrical contact was maintained between the corneal surface and the recording electrode by 2.5% methylcellulose (Goniosol; Ciba Vision). ERG was recorded from the left eye using a platinum wire electrode built into the tip of a fiber optic cable. A gold reference electrode was placed on the right eye. The light source was a 100 W halogen bulb (Xenophot HLX 64623; Osram) driven by a constant power source (ATE 15-15 M; Kepco Power Supplies). Stimuli consisted of either 2 or 10 ms light flashes of light (505 nm bandpass filter ± 17 nm, Andover Corp.). Flashes of varying intensities were delivered by attenuating the above light source with neutral density filters. To obtain the brightest light levels, a camera flash unit was used to deliver flashes corresponding to 6.5 log photons/μm^2^ and 8.75 log photons/μm^2^. Responses were averaged over 3–20 flashes for each flash intensity, with inter stimulus intervals ranging from 2.2 to 300 sec. Experiments were conducted over a total duration of 35–45 min. Light stimuli were calibrated daily using a photometer (Model 350 optical power meter, UDT instruments) and the results of the calibration informed calculations of light intensity.

Sensitivity was calculated as the I_1/2_ of the intensity-response curve when the a-wave data were fit to a modified Michaelis-Menten equation:

1 $$ \mathrm{R} = {\mathrm{R}}_{\max} \bullet {\mathrm{I}}^{\mathrm{n}}\ /\ \left({\mathrm{I}}^{\mathrm{n}}{{ + \mathrm{I}}_{\frac{1}{2}}}^{\mathrm{n}}\right) + \mathrm{base} $$

where R is the amplitude of the a-wave, R_max_ is the maximum amplitude of the a-wave, I is the flash energy (photons/μm^2^), I_1/2_ is the flash energy that elicits the a-wave amplitude of half R_max_ and n is an exponent, which is a dimensionless scaling parameter [47-49].

For photocurrent activation measurement, light intensities recorded as photons/μm^2^ incident at the cornea were converted to photoisomerizations/rod/second (Rh*) by using a corneal collection area of 0.102 μm^2^ for wild-type [50] and 0.091 μm^2^ for tg, based on an average outer segment length of 27.0 μm and 21.9 μm respectively. Data was amplified 2000×, AC filtered at 0.1 Hz high-pass and 300 Hz low-pass, digitized at 2 KHz and was analyzed by Labview (National Instruments) and IGOR PRO (Wavemetrics). To calculate phototransduction gain, we fit the leading edge of the a-wave up to the inflection point of the curve, using the model of phototransduction defined by the equation [27]:

2 $$ \mathrm{f}\left(\mathrm{t}\right) = \left\{1\ \hbox{--}\ \exp \left[\hbox{-} 0.5\kern0.5em \bullet \kern0.5em \upphi \kern0.5em \bullet \kern0.5em {\mathrm{A}}_{\mathrm{g}}{\left({\mathrm{t}\ \hbox{-}\ \mathrm{t}}_{\mathrm{delay}}\right)}^2\right]\ \right\}\kern0.5em \bullet \kern0.5em {\mathrm{a}}_{\max } $$

where f(*t*) is the a-wave response, is the flash intensity expressed in photoisomerizations per rod. A_g_ is phototransduction gain that scales flash intensity; t_delay_ is a brief delay due to the biochemical steps of the phototransduction activation process, and a_*max*_ is the maximum amplitude of the a-wave. For each animal, the rod model was fit to a family of responses to flash intensities from 2.96 to 5.4 log photons/μm^2^ incident light (90 – 25,000 R*/flash). Responses to flashes greater than 5.4 log photons/μm^2^ contained a cone component and thus were not used for curve fitting.

### Analysis of single cell and isolated retina tissue responses

For single cell suction electrode recordings, mice were dark adapted at least 3 hours prior to enucleation under IR illumination. Details of the recording procedure have been reported previously [51,52] with modification [53]. Gain calculations were only performed on cells in which single photon responses were measured, from which collecting areas (Ac) were calculated (A_c_ range: WT = 0.105 ± 0.014 μm^2^, n = 20; tg = 0.085 ± 0.019 μm^2^, n = 13; p 0.3). Gain calculations were performed using a modified Equation 2.

3 $$ \mathrm{f}(t) = \left\{1\hbox{-} \exp \left[\hbox{-} 0.5\bullet {\mathrm{A}}_{\mathrm{g}}\bullet {\mathrm{A}}_{\mathrm{c}}\bullet \upphi \bullet {\left({\mathrm{t}\hbox{-} \mathrm{t}}_{\mathrm{delay}}\right)}^2\right]\right\} \bullet {\mathrm{R}}_{\max } $$

Where f(t) is the single cell response, A_g_ is phototransduction gain in s^−2^, A_c_ is the collecting area of the cell in μm^2^, derived from the single photon response [51], and ϕ is the photon density of the flash that elicited the response, in photons/μm^2^. The single photon response was measured without assumptions of collecting area. The variance to mean ratio was calculated for a large series of responses to brief flashes (typically 40–50) of dim light. From this analysis, along with the calibration of the light stimuli for each individual cell, the collecting area for each cell was calculated. This collecting area value was applied to all stimuli to estimate numbers of absorbed photons, which was used to estimate I_1/2_ and in the gain-fitting equation 3. For isolated retinal tissue, mice were dark adapted at least 3 hours prior to enucleation under IR illumination. The retina was isolated in HEPES buffer containing 10 mM BaCl_2_, and retina was embedded in a grease ring, photoreceptor side up, and held in place by a circular piece of filter paper with a 2 mm diameter hole in the center. Electrical connectivity was made with two silver chloride pellets; one embedded in the chamber adjacent to the nerve fiber layer, and the second adjacent to the solution flowing over the tissue. The retina was perfused with a 37°C bicarbonate buffered Locke’s solution that included 10 mM D/L aspartate and 25 μM (2S)-2-amino-4-phosphonobutanoic acid (AP-4). These selective inhibitors blocked transmission of the photoreceptor signal to the on-bipolar cells. Flow rate was held at 1 ml/minute. Additionally, the bath reservoir was bubbled with a 95/5 mixture of O_2_/CO_2_ to maintain the pH of the perfusion solution at 7.5. Data was collected as in ERG recordings. Gain fits were performed only on responses to dim flashes that contained no apparent cone components.

### Statistical analysis

Two-tailed Student’s t-tests performed in Excel 2010 were used to determine statistical significance in all comparisons unless otherwise noted. Error is expressed as standard error of the mean or standard deviation as noted in the text. Analysis of rhodopsin concentration required two-way ANOVA performed in MS Excel 2010.

## Additional files

Additional file 1: Figure S1. Localization of GARP2 with C-terminal GARP2-specific antibodies in **(A)** WT and **(B)** tg mouse retina. Antibodies (GARP-C, see Methods) were generated against the unique 8 amino acid C-terminal sequence of GARP2 (RATAAGGL) and used for immunohistochemistry. GARP2 predominantly localizes in ROS in both WT and tg retinas. Minor labeling is also apparent in the outer plexiform layer in the tg retina which probably reflects the overexpression of GARP2 in the transgenic mouse as the ROS labeling also appears more intense in the tg retina. Additional file 2: Figure S2. ERG amplitudes for WT (black) and tg (red). **(A)** average a-wave responses with increasing stimulus intensity. The maximum a-wave in the tg animals was decreased compared to WT, likely due to shortened outer segments. **(B)** a-wave responses fit with equation (1) to estimate photoreceptor sensitivity. WT and tg responses are fit with similar I_1/2_ value. **(C-D)** same as **(A-B)** for b-wave amplitudes. Additional file 3: Figure S3. Measurement of disk spacing across three disks in **(A)** WT (n = 4) and **(B)** tg (n = 5) mouse rod outer segment TEM images. Retina EM samples photographed at original magnifications of 75,000 to 200,000 were used to measure the distance across three complete disks in five locations on each image. **(A)** WT retina showing one region of rod outer segment used for measuring (orig. mag. 75,000, scale bar 500 nm). Inset shows an enlarged image of the boxed segment below. **(B)** tg retina showing a region of rod outer segment (orig. mag. x75,000, scale bar 500 nm). Inset shows one enlarged region used for measuring that is boxed below. **(C)** Individual measurements for each image were averaged and the average of all disk measurements for each genotype is shown in the bar graph. Error is reported in standard deviation. No significant difference (p > 0.16) in disk spacing was observed.

## Abbreviations

A_c_: Collecting area of the cell
A_g_: Measure of phototransduction gain
CNG: Cyclic nucleotide gated
CNGA1: Rod cyclic nucleotide gated cation channel α-subunit
CNGB1: Rod cyclic nucleotide gated cation channel β-subunit
ERG: Electroretinography
GARP: Glutamic acid rich protein
GC1: Rod guanylate cyclase I
tg: Mouse strain containing transgene overexpressing GARP2
ONH: Optic nerve head
ONL: Outer nuclear layer
PDE6: 3′, 5′ cGMP phosphodiesterase type 6
PDE6G: 3’, 5’ cGMP-phosphodiesterase type 6 γ-subunit
PER2: Peripherin2/RDS protein
ROS: Rod outer segment
spr: single photon response amplitude
T*: Activated transducin
T_i_: Integration time
T_p_: Time-to-peak
τ_D_: Dominant time constant
