# Supplementary figures and images for: Overexpression of rod photoreceptor glutamic acid rich protein 2 (GARP2) increases gain and slows recovery in mouse retina
Source: Cell Commun Signal. 2014 Oct 17;12:67. doi: 10.1186/s12964-014-0067-5 (PMC4207353; doi:10.1186/s12964-014-0067-5)

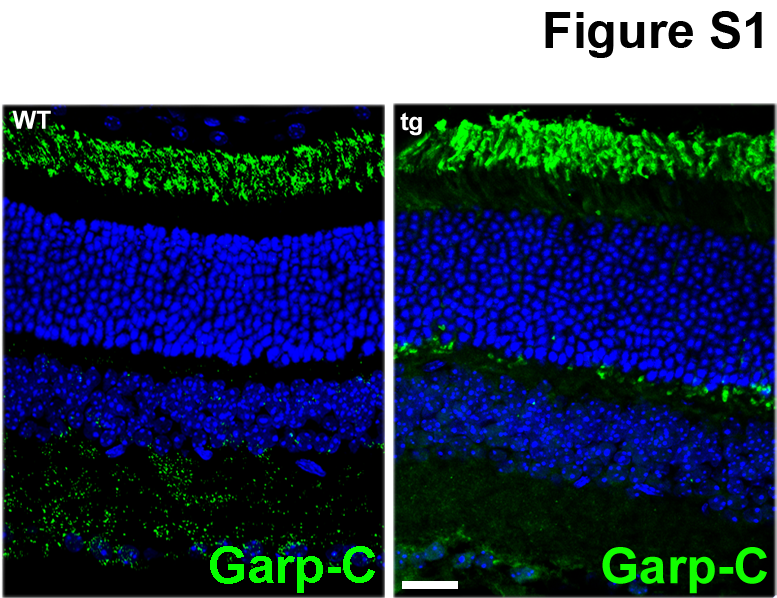

Supplement: Additional file 1: Figure S1. — Localization of GARP2 with C-terminal GARP2-specific antibodies in (A) WT and (B) tg mouse retina. Antibodies (GARP-C, see Methods) were generated against the unique 8 amino acid C-terminal sequence of GARP2 (RATAAGGL) and used for immunohistochemistry. GARP2 predominantly localizes in ROS in both WT and tg retinas. Minor labeling is also apparent in the outer plexiform layer in the tg retina which probably reflects the overexpression of GARP2 in the transgenic mouse as the ROS labeling also appears more intense in the tg retina. [file 12964_2014_67_MOESM1_ESM.tiff]

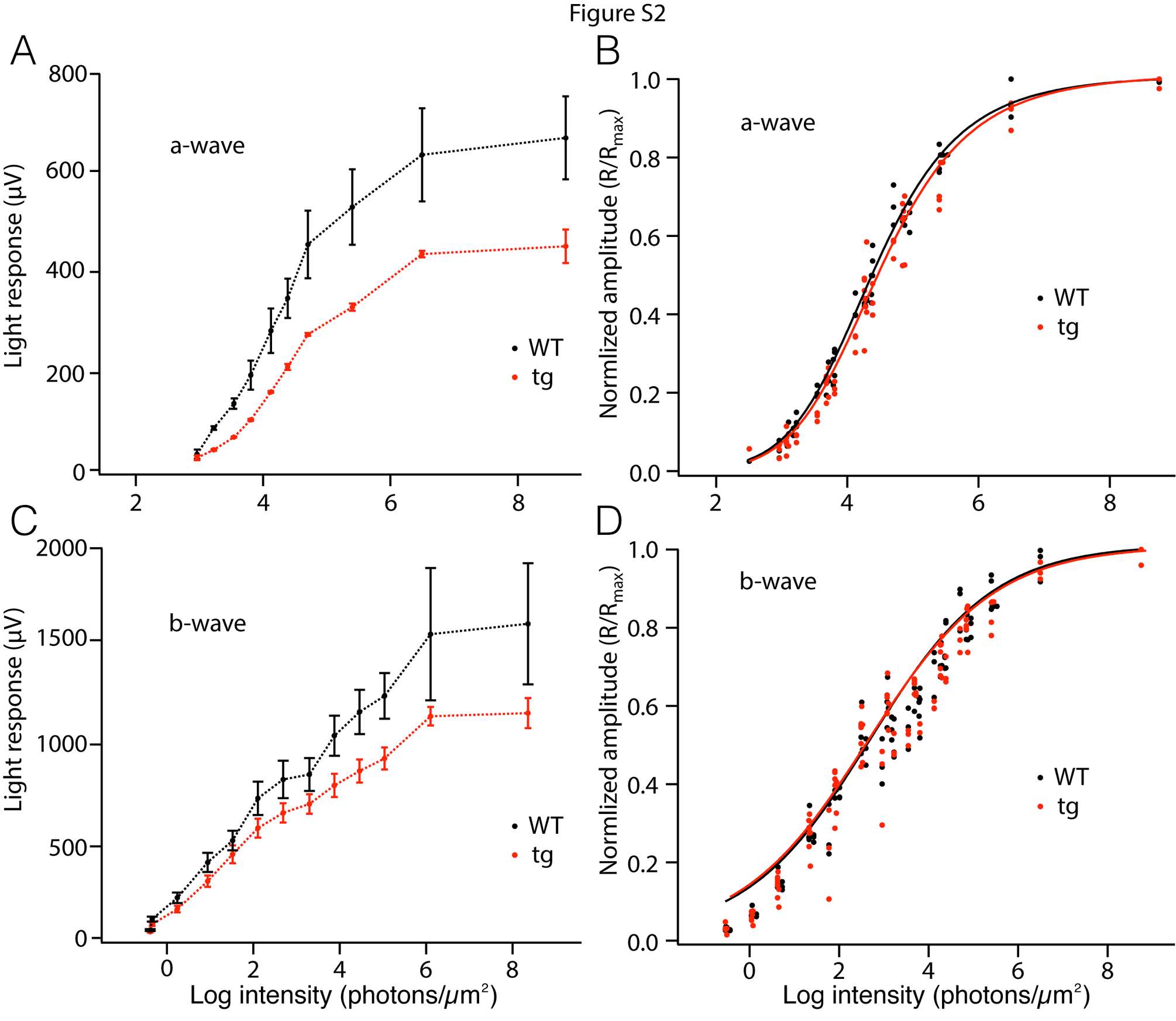

Supplement: Additional file 2: Figure S2. — ERG amplitudes for WT (black) and tg (red). (A) average a-wave responses with increasing stimulus intensity. The maximum a-wave in the tg animals was decreased compared to WT, likely due to shortened outer segments. (B) a-wave responses fit with equation (1) to estimate photoreceptor sensitivity. WT and tg responses are fit with similar I1/2 value. (C-D) same as (A-B) for b-wave amplitudes. [file 12964_2014_67_MOESM2_ESM.tif]

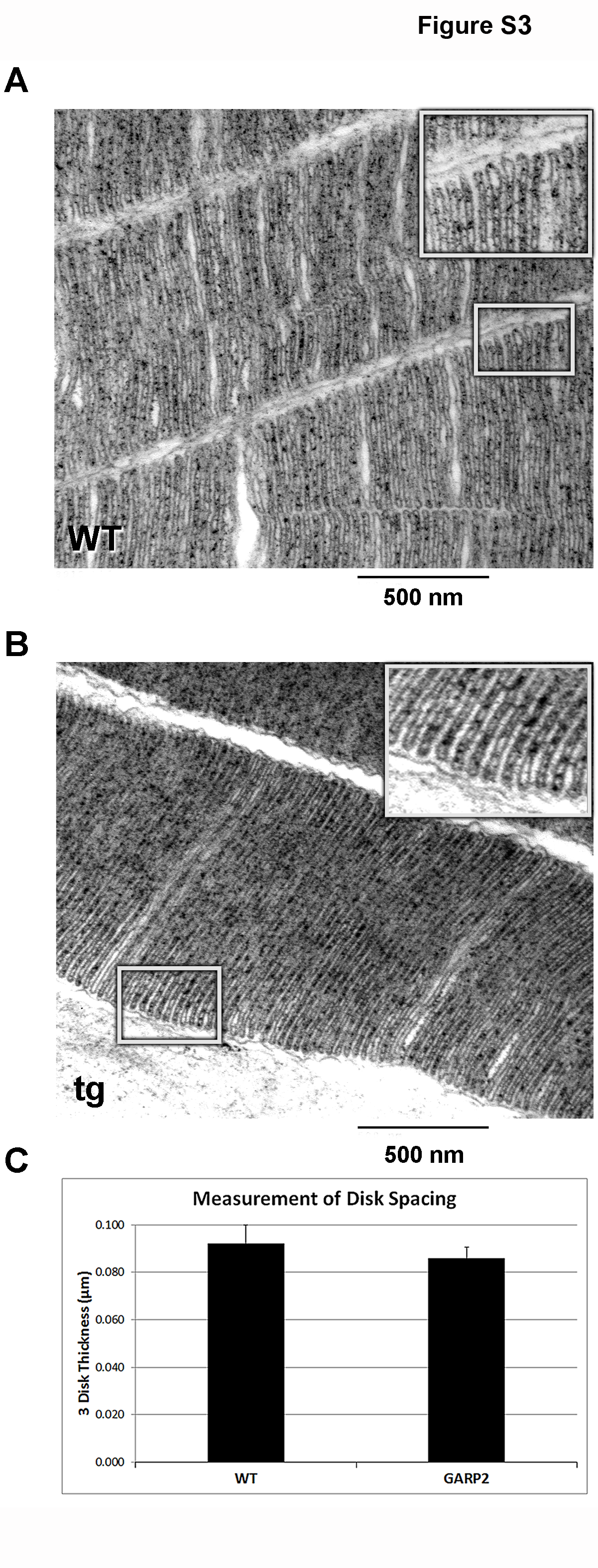

Supplement: Additional file 3: Figure S3. — Measurement of disk spacing across three disks in (A) WT (n = 4) and (B) tg (n = 5) mouse rod outer segment TEM images. Retina EM samples photographed at original magnifications of 75,000 to 200,000 were used to measure the distance across three complete disks in five locations on each image. (A) WT retina showing one region of rod outer segment used for measuring (orig. mag. 75,000, scale bar 500 nm). Inset shows an enlarged image of the boxed segment below. (B) tg retina showing a region of rod outer segment (orig. mag. x75,000, scale bar 500 nm). Inset shows one enlarged region used for measuring that is boxed below. (C) Individual measurements for each image were averaged and the average of all disk measurements for each genotype is shown in the bar graph. Error is reported in standard deviation. No significant difference (p > 0.16) in disk spacing was observed. [file 12964_2014_67_MOESM3_ESM.tiff]
